# Strigolactones inhibit auxin feedback on PIN-dependent auxin transport canalization

**DOI:** 10.1038/s41467-020-17252-y

**Published:** 2020-07-14

**Authors:** Jing Zhang, Ewa Mazur, Jozef Balla, Michelle Gallei, Petr Kalousek, Zuzana Medveďová, Yang Li, Yaping Wang, Tomáš Prát, Mina Vasileva, Vilém Reinöhl, Stanislav Procházka, Rostislav Halouzka, Petr Tarkowski, Christian Luschnig, Philip B. Brewer, Jiří Friml

**Affiliations:** 1 State Key Laboratory of Plant Physiology and Biochemistry, College of Biological Sciences, China Agricultural University, Beijing, 100193 China; 2 University of Silesia in Katowice, Faculty of Natural Sciences, Institute of Biology, Biotechnology and Environmental Protection, Jagiellońska 28, 40-032 Katowice, Poland; 3 Mendel Centre for Plant Genomics and Proteomics, Central European Institute of Technology (CEITEC), Masaryk University (MU), 62500 Brno, Czech Republic; 4 Central European Institute of Technology (CEITEC), Mendel University in Brno, Zemedelska 1, 61300 Brno, Czech Republic; 5 Department of Plant Biology, Mendel University in Brno, Zemedelska 1, 61300 Brno, Czech Republic; 6 Institute of Science and Technology (IST), Klosterneuburg, 3400 Austria; 7 Central Laboratories and Research Support, Centre of the Region Haná for Biotechnological and Agricultural Research, Palacký University, Šlechtitelů 27, 78371 Olomouc, Czech Republic; 8 Department of Applied Genetics and Cell Biology, University of Natural Resources and Life Sciences, Vienna (BOKU), Muthgasse 18, 1190 Wien, Austria; 9 ARC Centre of Excellence in Plant Energy Biology, School of Agriculture, Food and Wine, Waite Research Precinct, The University of Adelaide, Glen Osmond, SA 5064 Australia

**Keywords:** Plant cell biology, Plant development, Plant hormones

## Abstract

Directional transport of the phytohormone auxin is a versatile, plant-specific mechanism regulating many aspects of plant development. The recently identified plant hormones, strigolactones (SLs), are implicated in many plant traits; among others, they modify the phenotypic output of PIN-FORMED (PIN) auxin transporters for fine-tuning of growth and developmental responses. Here, we show in pea and *Arabidopsis* that SLs target processes dependent on the canalization of auxin flow, which involves auxin feedback on PIN subcellular distribution. D14 receptor- and MAX2 F-box-mediated SL signaling inhibits the formation of auxin-conducting channels after wounding or from artificial auxin sources, during vasculature de novo formation and regeneration. At the cellular level, SLs interfere with auxin effects on PIN polar targeting, constitutive PIN trafficking as well as clathrin-mediated endocytosis. Our results identify a non-transcriptional mechanism of SL action, uncoupling auxin feedback on PIN polarity and trafficking, thereby regulating vascular tissue formation and regeneration.

## Abstract (web summary)

Strigolactones are a newly identified, but incompletely characterized class of plant hormones play crucial roles in plant development. Here the authors show that strigolactones prevent an auxin feedback-effect on PIN-FORMED (PIN) polarity and trafficking, thereby regulating vascular tissue formation and regeneration.

## Introduction

Plant development is characterized by self-organizing processes, such as the regular patterns of organ initiation at the shoot apical meristem, branching of roots and shoots, the connection of newly formed organs with pre-existing vasculature, or the spontaneous occurrence of vasculature veins in developing leaves. The plant hormone auxin and its directional transport through tissues have been implicated in all these traits^1^. The process that is called auxin canalization establishes narrow auxin transport routes between cells and tissues of relatively high auxin concentration (source), to locations where auxin is being depleted (sink)^2–4^. A self-reinforcing system has been proposed to drive canalization. In this system, auxin feeds back on PIN-FORMED (PIN) auxin transporters by promoting the expression of *PIN* genes specifically in auxin transport routes and by localizing PINs to plasma membrane (PM) domains facing the auxin sink^3,5^. Auxin is typically transported basipetally from source to sink and canalization seems to be driven by auxin sink rather than source^6–8^. For example, developing vasculature tissue is characterized by relatively high auxin contents; therefore, sink strength in such a system primarily depends on PIN-dependent auxin flux rates depleting auxin from sources. New vein patterns in leaves develop away from a localized sink at the base, opposite to the direction of auxin flow^6–8^. Vasculature formation and its connection to already existing vascular strands hence is intimately linked to the effects of auxin flux on the subcellular positioning of PINs, which in turn defines auxin flux rates and directionality^6–8^. Related feedback mechanisms controlling PIN polarity have been described for additional developmental processes, such as embryonic axis formation^9,10^, shoot and root organogenesis^11,12^ as well as the control of the directional growth of organs^13^.

In spite of the biological significance of PIN proteins function, the mechanisms by which auxin controls polarization of PINs have remained conceptually unclear. Modeling of auxin-mediated polarization^14^ has linked auxin feedback on PIN polarity to the auxin effect on PIN subcellular trafficking^15–17^. PM-associated PINs are internalized in clathrin-coated vesicles in a process called endocytosis^18–20^ that might precede PIN relocation to different (plasma) membrane domains^21,22^. Numerous pharmacological and genetic determinants that impact on specific cellular events in the control of auxin transport have shaped our current picture of cellular mechanisms and crosstalk therein^1^. For example, treatment with the fungal toxin Brefeldin A (BFA) revealed the constitutive endocytic recycling of PIN proteins. This requires BFA-mediated interference of GNOM ARF-GEF activity, causing PM proteins, including PINs, to aggregate in cells^18,20^. Notably, auxin itself appears to inhibit the process of endocytosis and antagonize the BFA effect on PIN recycling^15^. In addition to auxin effects on PINs, various other plant hormones can influence PIN-dependent auxin transport, such as cytokinins^23^, brassinosteroids^24,25^, gibberellins^26^, salicylic acid^27^, abscisic acid^28^, and strigolactones (SLs)^29^. However, much remains to be uncovered about the modes of action, by which these plant hormones regulate PINs, thereby ultimately defining plant development.

SLs represent a recently discovered class of plant growth regulators and their developmental roles and signaling mechanisms are not yet fully characterized. SLs have been shown to influence a range of plant traits including shoot branching^30,31^, shoot gravitropism^32^, secondary growth^33^, adventitious rooting^34^ as well as lateral rooting and root hair elongation^35,36^. Many of the processes targeted by SLs also require auxin transport, specifically its canalization as proposed for the classical SL effect on shoot branching^37^. This response has been linked to SL-mediated interference of PIN PM targeting and the modulation of auxin flux^29,37^, but we still lack insight into the mechanisms by which SLs might impact the sorting of PINs.

Our observations in this study extend the spectrum of physiological SL effects on processes associated with auxin transport canalization, namely leaf venation and vascular tissue regeneration and formation induced by wounding or external auxin sources. At the cellular level, we show that SLs specifically interfere with the feedback of auxin on PIN polarization and clathrin-mediated internalization, providing a mechanistic framework for the molecular action of SLs in many developmental processes.

## Results

### SLs interfere with auxin canalization in pea

Inhibition of shoot branching is among the best-understood responses of SLs in flowering plants. This is a process that involves auxin canalization, because when buds are released from dormancy, they initiate the formation of PIN1-expressing channels to increase vascular connections with the main vasculature^5^. These channels appear similar to those that form after adding exogenous auxin to the side of the stem^3,5^. These canalization events can be inhibited by auxin produced in shoot apices, hinting at a possible mechanism, by which dominant shoot tips might control branching^5^. When SLs are applied directly to buds after decapitation, they inhibit bud outgrowth^38^ and reduce the transport of auxin (indole-3-acetic acid, [^3^H]-IAA) from buds into the stem (Supplementary Fig. 1a–d). However, the precise action of SLs in controlling auxin canalization and vascularization is less obvious^39^. Therefore, we explored the effect of synthetic SL, *rac*-GR24 (hereafter called GR24) using intact or fully decapitated pea (*Pisum sativum*) plants that had been treated with auxin (indole-3-acetic acid; IAA).

**Fig. 1:**
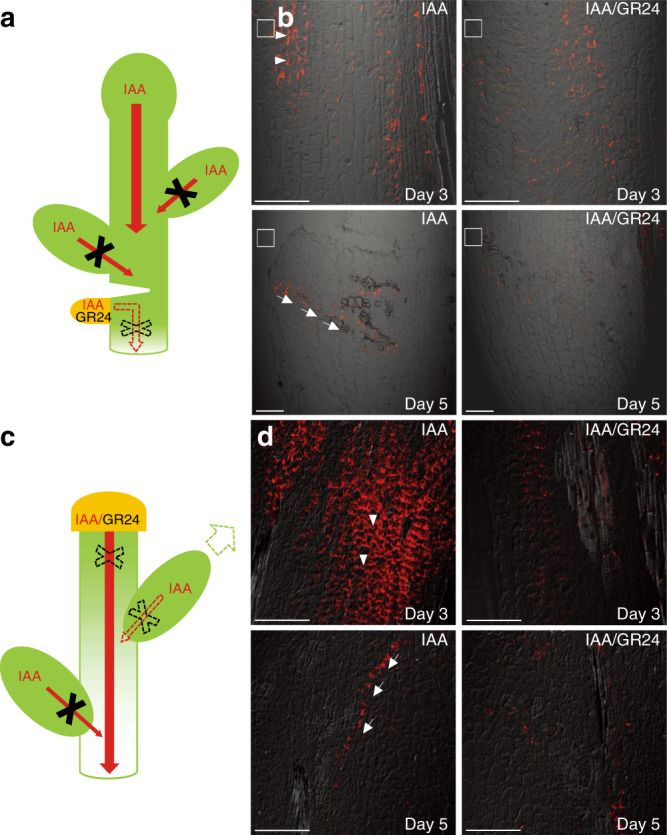
SL effects on PIN-dependent auxin canalization in pea. **a** Scheme representing plants wounded below the lower buds. Red arrows represent auxin (IAA) flow. Red arrows crossed with black X represent inhibited auxin flow. Lanolin pastes containing IAA or IAA/GR24 (marked in yellow) were applied to the side of the pea stem below the wound. Dashed red arrow crossed with dashed black X represents formation of a new auxin flow route from the auxin source that can be inhibited by GR24 application. **b** Immunolocalization of PIN1 in the primary stem. The concentrations of IAA and GR24 applied locally in lanolin pastes were 0.16 and 0.09 µM, respectively. In total, 10 plants were analyzed for each treatment. White rectangles indicate the places of IAA or IAA/GR24 application. Arrowheads indicate polarity of the PIN localization. Arrows indicate newly formed auxin channels. The fluorescence signals were evaluated on Olympus Fluoview 200 confocal scanning microscope with UPlanFI 20×/0.5 and/or UPlanApo 10×/0.40 objectives. PIN1 immunolocalization signals (red) are overlaid with the transmitted light images. Scale bar, 100 μm. **c** Scheme of decapitated plants treated with IAA or IAA/GR24 paste on the stump. Red arrows represent auxin (IAA) flow. Red arrow crossed with dashed black X represents formation of a new auxin flow route from the auxin application to the stump that is inhibited by GR24 application. Dashed red arrow crossed with dashed black X represents intermitted auxin flow after temporary bud activation. Dashed green arrow represents temporary bud outgrowth. Red arrows crossed with black X represent inhibited auxin flow. **d** Immunolocalization of PIN1 in the stem of decapitated plants. In total, 10 plants were analyzed for each treatment. Arrowheads indicate polarity of the PIN localization. Arrows indicate newly formed auxin channels. The fluorescence signals were evaluated on Olympus Fluoview 200 confocal scanning microscope with UPlanFI 20×/0.5 objective. PIN1 immunolocalization signals (red) are overlaid with the transmitted light images. Scale bar, 100 μm. The above experiments were repeated three times with similar results. Images shown are representative of each treatment.

First, we analyzed PIN1 channel and subsequent vasculature formation originating from an artificial lateral auxin source. Lateral, local auxin application in lanolin paste onto pea stems just below a cut (Fig. 1a) was sufficient to induce the formation of PIN1-expressing auxin channels and subsequent vascular connections to the stem vasculature^4,40^. In our control situation, strong PIN1 expression in the vicinity of the local IAA application was observed with a predominantly lateral PIN1 localization, pointing away from the auxin source (Fig. 1b). An initially large area of PIN1-expressing cells narrowed down about 5 days after auxin application, resulting in the establishment of fully defined and strongly polarized narrow PIN1 channels, often accompanied by differentiated xylem vessels (Fig. 1b). This is in agreement with the classical canalization hypothesis in the absence of competing auxin sources^2^. In contrast, co-application of GR24 interfered with strongly polarized PIN1 expression as well as with the formation of PIN1 channels and continuous de novo vasculature; only occasional fragmented xylem cells appeared instead (Fig. 1b).

Related observations were made when we analyzed PIN1 expression in fully decapitated pea stems. IAA application to the stump (Fig. 1c) led to a massive increase of PIN1 expression in the polarized field below the application site within the first 3 days, while the formation of narrow PIN1-expressing channels accompanied with differentiated xylem strands became visible after 5 days (Fig. 1d). The simultaneous application of GR24 strongly inhibited this process, as we failed to observe a pronounced increase in polarized PIN1 expression and channel formation under these conditions (Fig. 1d).

Together, our findings suggest an inhibitory role of SLs in the formation of new auxin-conducting, PIN-expressing channels induced from auxin sources. This effect of SLs on auxin canalization would offer a plausible explanation for how SLs regulate auxin transport, vascularization and branching.

### SLs inhibit vasculature formation and regeneration

**Fig. 2:**
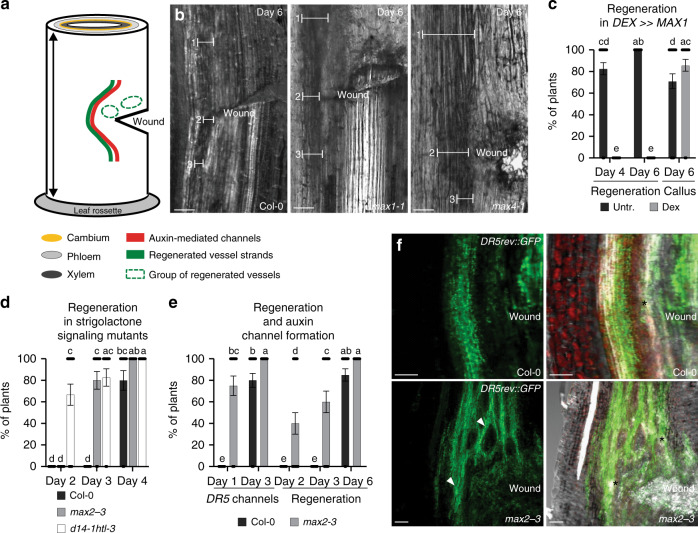
SL regulation of vasculature regeneration after wounding in *Arabidopsis* stems. **a** Scheme representing spatial changes around a wound during vascular tissue regeneration in incised stems of *Arabidopsis*. Wounding is made in the basal part of inflorescence stem just above the rosette leaves to disturb the longitudinal continuum of the vascular cambium. Green line represents development of regenerated vessel strands around a wound. Red line represents auxin-mediated channels formation. Green circles represent the groups of vessel-like cells developed from outer cortex or callus in the neighborhood of the wound. **b** Vascular tissue regeneration in SL biosynthesis defective mutants *max1-1* and *max4-1*. Line segments indicate the thickness of regenerated vasculature; above the wound (1), close to the wound (2), below the wound (3). Scale bars: 100 µm. **c** Vascular tissue regeneration in wounded *DEX*≫*MAX1* plants. Data are expressed as mean ± s.e.m. (*n* ≥ 22 inflorescence stems). Means with different letters are significantly different at *P* < 0.05 (one-way ANOVA with Fisher LSD test). **d** Vascular tissue regeneration in SL/karrikin signaling-defective mutants *max2-3* and *d14-1 htl-3*. Data are expressed as mean ± s.e.m. (*n* ≥ 15 inflorescence stems). Means with different letters are significantly different at *P* < 0.05 (one-way ANOVA with Fisher LSD test). **e**, **f** The formation of auxin channels around a wound as inferred from *DR5rev::GFP* expression during vascular tissue regeneration. Data are expressed as mean ± s.e.m. (*n* ≥ 24 inflorescence stems). Means with different letters are significantly different at *P* < 0.05 (one-way ANOVA with Fisher LSD test). Right panels in **f** are merged images of fluorescence and light transmitted signals. Arrowheads indicate abundant channels. Asterisks indicate regenerated vasculature. Scale bars: 100 µm. The above experiments were repeated twice with similar results. Images shown are representative of each treatment. Source data of **c**–**e** are provided in the Source data file.

To further explore the role of SLs in other processes that have been mechanistically linked to canalization, we examined canalization-dependent vasculature regeneration following wounding^2^, which has recently been established in *Arabidopsis* (*Arabidopsis thaliana*) stems^41^ (Fig. 2a). This allowed us to use the extensive genetic toolkit in the model species, and also allowed us to test plant-produced, endogenous SLs rather than relying on synthetic SLs.

In control experiments, we observed vascular regeneration initiated with a broad PIN1 expression field and auxin accumulation above the wound about 2 days after wounding. This was subsequently followed by the establishment of narrow, polarized PIN1-expressing, auxin-conducting channels circumventing the wounded site^41^ during the next days (Supplementary Fig. 2a). Strikingly, vasculature regeneration in the SL biosynthesis mutants *more axillary growth (max)1-1* and *max4-1* occurred as fast as in control (Supplementary Fig. 2b), but the regenerated vasculature in the mutants was more abundant than that in the wild-type control (Fig. 2b). To test the effect of increased endogenous SLs, we employed conditional overexpression of the cytochrome P450 monooxygenase *MAX1* in the *max1* mutant background (*DEX*≫*MAX1 max1-1*; hereafter named *DEX*≫*MAX1*). In un-induced control plants, the first vessels around the wound appeared after 4 days and fully regenerated vasculature was observed after 6 days (Fig. 2c, Supplementary Fig. 2c). In contrast, although we detected clusters of isolated vessel-like cells that developed from callus in both untreated and dexamethasone (Dex)-treated plants (Fig. 2c), there was no regeneration of vasculature around the wound observed after Dex induction (Fig. 2c, Supplementary Fig. 2c). These results are in line with the observations that we made in pea, substantiating an inhibitory role for SLs in the regulation of canalization-mediated vasculature regeneration.

We also determined the efficiency of vasculature regeneration in mutants affected in SL/karrikin-related signaling, including a mutant allele affected in the MAX2 F-box protein (*max2-3*) and a double mutant affected in SL/karrikin receptors *dwarf14-1 hyposensitive to light-3* (*d14-1 htl-3*)^42^. In both genotypes, regeneration occurred faster (Fig. 2d) and the regenerated strands were more abundant compared with wild-type control (Supplementary Fig. 2d), suggesting that SL/karrikin signaling normally restricts vasculature regeneration.

To directly assess whether SL/karrikin signaling is involved in auxin channel formation, we analyzed the expression of the *DR5* auxin response reporter (*DR5rev::GFP*) during regeneration. Comparison between wild type and *max2-3* revealed that the *DR5*-positive channels formed faster and more abundantly when SL/karrikin signaling was compromised (Fig. 2e, f). Consistently, the layer of regenerated vasculature was also formed earlier and thicker in the *max2-3* mutant (Fig. 2e, f).

Together, these results identify SLs as crucial regulators of vasculature regeneration after wounding, and that increased SL levels inhibit, whereas decreased SL biosynthesis or compromised SL signaling promotes, canalization-mediated vasculature regeneration. Another presumably auxin canalization-dependent process that involves vasculature patterning along auxin channels is de novo leaf venation formation^2,6,8,43^. We questioned whether SLs might participate in this process as well, and thus examined leaf vascular development in presence of GR24 or upon induction of endogenous SL biosynthesis. After growth on GR24, simplified leaf vascular network patterns with occasional discontinuities were detected (Supplementary Fig. 3a, b). Dex-treated *DEX*≫*MAX1* plants also caused more simplified leaf veins with more free ends (Supplementary Fig. 3c, d). This two-component glucocorticoid system can occasionally cause non-specific growth defects^44^. However, our Dex-treated transgenic plants expressing only the chimeric GAL4-VP16-GR (GVG) transcription factor grew normally and leaf vasculature was unaffected (Dex: 11.1 free-ending veins per leaf, *n* = 20 leaves; Control: 10.7 free-ending veins per leaf, *n* = 20 leaves). Therefore, these data support the notion that SLs regulate vasculature regeneration as well as de novo formation during venation patterning in leaves.

### SLs interfere with auxin-mediated PIN polarization

**Fig. 3:**
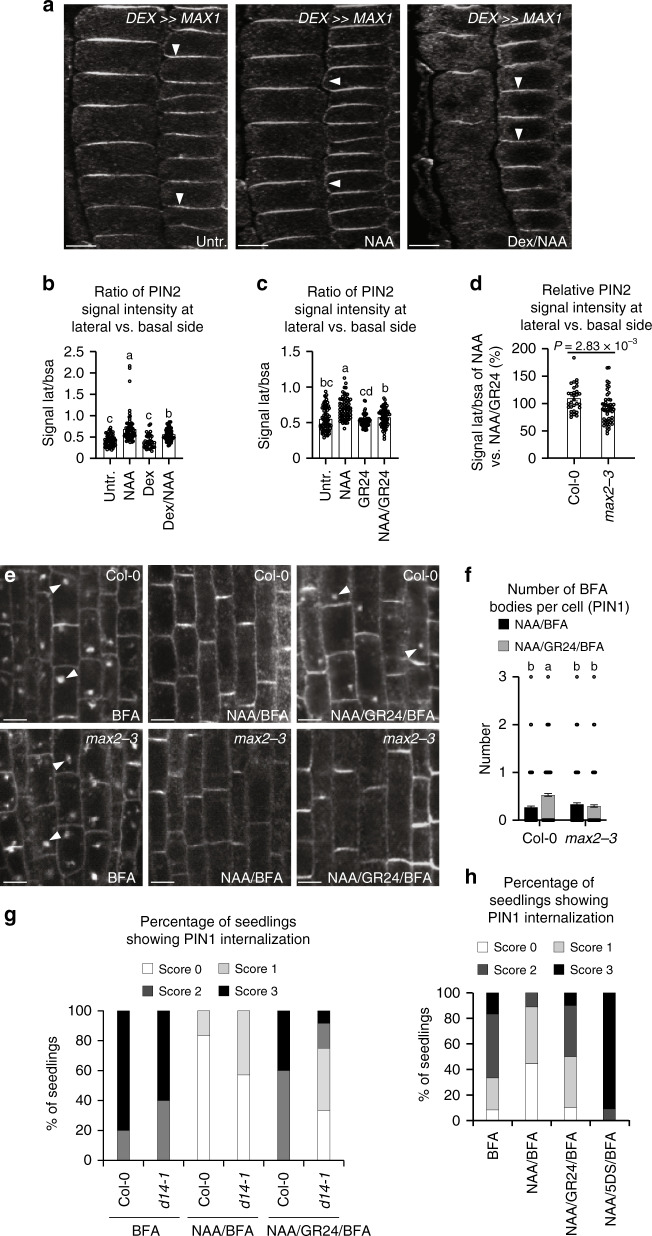
SL effect on auxin-regulated PIN subcellular dynamics. **a**, **b** Endogenous SL effect on auxin-mediated PIN2 polarity changes in young cortex cells. Immunolocalization of PIN2 is shown (**a**). Arrowheads indicate the polarity of PIN localization. Scale bars: 5 µm. Ratio between mean fluorescence intensity of the lateral and basal membrane in young cortex cells was scored (**b** *n* ≥ 34 cells). **c** Exogenous SL effect on auxin-mediated PIN2 polarity changes in young cortex cells. Ratio between mean fluorescence intensity of the lateral and basal membrane in young cortex cells was scored (**c** *n* ≥ 70 cells). Data are expressed as mean ± s.e.m. Means with different letters are significantly different at *P* < 0.05 (one-way ANOVA with Fisher LSD test) (**b**, **c**). **d** Less pronounced effect in *max2-3* SL/karrikin signaling mutant in terms of GR24 inhibition of NAA action on PIN2 lateralization (**d** *n* ≥ 30 cells). Data are expressed as mean ± s.e.m. *P* value was calculated using Welch’s two-tailed *t*-test. **e**, **f** GR24 effect on NAA inhibition of BFA-induced PIN1 internalization. Immunolocalization of PIN1 in root cells is shown (**e**). Arrowheads indicate PIN1 proteins internalized into BFA compartments. Scale bars: 5 µm. The number of BFA bodies per root cell in NAA/BFA- or NAA/GR24/BFA-treated wild-type and *max2* seedlings was scored (**f** *n* ≥ 425 cells). Data are expressed as mean ± s.e.m. Means with different letters are significantly different at *P* < 0.05 (one-way ANOVA with Fisher LSD test). **g**, **h** Quantification of PIN1 internalization in roots. Both synthetic SL GR24 (25 μM, **g**; 50 μM, **h**) and natural SL 5DS (50 μM, **h**) were applied. The same position of root tip was imaged with the same microscope settings for each independent experiment. The roots (*n* ≥ 5) were then scored blind and the percentage of roots displaying almost undetectable (Score 0), weak (Score 1), stronger (Score 2), or very severe (Score 3) PIN1 internalization was determined. The above experiments were repeated three times with similar results. Images shown are representative of each treatment. Source data of **b**–**d** and **f**–**h** are provided in the Source data file.

The mechanism by which a local auxin source promotes the formation of auxin channels and vascularization is largely unknown. The classical canalization hypothesis proposes positive auxin feedback on auxin transport directionality^14^, which can be realized at the cellular level by the effect of auxin on PIN polar distribution. This can be visualized by auxin-mediated PIN polarity rearrangements in *Arabidopsis* roots^4,45^. In primary roots, PIN2 localizes to the apical side of epidermal cells, and preferentially to the basal cell side in the young cortex cells^22^. Auxin (synthetic 1-naphthaleneacetic acid; NAA or natural; IAA) treatments led to rearrangement in PIN2 distribution to the outer lateral sides of cortex cells^4^ (Fig. 3a–c). This PIN lateralization effect of auxin was consistently attenuated by induction of SL biosynthesis in the *DEX*≫*MAX1* line (Fig. 3a, b), and by NAA/GR24 co-treatment in wild-type seedlings (Fig. 3c). In addition, we tested the effect of a natural SL, (+)-5-deoxystrigol (5DS), on NAA-mediated PIN2 lateralization and observed an inhibitory response similar to that of GR24 (Supplementary Fig. 6c). In contrast, the GR24 effect on auxin-mediated PIN2 lateralization was diminished in *max2-3* (Fig. 3d).

These observations suggest that SLs, acting via a MAX2-dependent signaling pathway, not only negatively regulate the canalization processes at the organ and tissue levels, but also auxin-mediated polarization of PIN transporters at the level of individual cells.

### SLs interfere with auxin effect on PIN endocytic recycling

How auxin can regulate PIN polarity and, in particular, how a localized auxin source can lead to the coordinated polarity changes in a whole field of cells, is conceptually unclear. Modeling of canalization and PIN polarization suggests that auxin feedback on PIN polarity can be related to the known inhibitory auxin effect on PIN internalization in individual cells^14^. PIN proteins are known to constitutively cycle between the PM and the endosomes^18,46^. This cycling is sensitive to BFA^18^, which preferentially inhibits PIN trafficking to the PM^22,47^, leading to the intracellular accumulation of constitutively cycling PIN proteins^18^. Previous studies have shown that PIN endocytosis and constitutive recycling are important in determining PIN polarity^48–50^, and intracellular PIN accumulation is rapidly and transiently inhibited by auxin itself^15^.

We investigated the SL effect on auxin-mediated inhibition of PIN endocytic recycling. As shown previously^15^, PIN proteins accumulated intracellularly after BFA treatment and such internalization was inhibited by NAA (Fig. 3e). GR24 treatment showed no effect on BFA-induced PIN intracellular accumulation (Supplementary Fig. 4a, b), but it clearly interfered with NAA-mediated inhibition of PIN internalization. This was reflected by increased accumulation of PIN1 and PIN2 in BFA-induced compartments, upon co-treatment with NAA/GR24 (Fig. 3e, f, Supplementary Fig. 4c–e). Similarly, 5DS also interfered with the auxin effect on the BFA-induced PIN intracellular accumulation (Fig. 3h, Supplementary Fig. 4f).

Note that some of these short-term pharmacological experiments required high concentrations of *rac*-GR24. High GR24 concentrations may impact on photoreceptor pathways^51^ and the use of *rac*-GR24 may lead to non-SL responses due to stereoisomer specificity^52^. However, 5 μM GR24 or higher can be required to trigger responses, particularly in roots^37,53–55^. We aimed to resolve these issues by testing the transgenic line *DEX*≫*MAX1* that stimulates endogenous SL biosynthesis^29^, and also comparing GR24 treatment responses with SL mutants. The same antagonistic SL effect on auxin-mediated inhibition of PIN internalization was observed in Dex-treated *DEX*≫*MAX1* line (Supplementary Fig. 4g–k). Furthermore, we tested whether the effect of SLs on PIN trafficking depends on SL signaling components. In the absence of GR24, BFA-induced PIN internalization or NAA-mediated inhibition was similar in the *max2* SL/karrikin signaling mutant or the *d14* SL-specific signaling mutant as that of the wild type (Fig. 3e–g, Supplementary Fig. 4d, e). Importantly, these mutants showed less sensitivity to GR24 in counteracting the NAA action on PIN endocytic trafficking (Fig. 3e–g, Supplementary Fig. 4d, e), which appeared consistent with the results from other pharmacological studies.

In summary, our findings imply that synthetic or endogenous SLs interfere with the antagonistic auxin effect on BFA-induced intracellular accumulation of PINs, by acting via D14- and MAX2-dependent SL signaling.

### SLs interfere with auxin effect on endocytosis

**Fig. 4:**
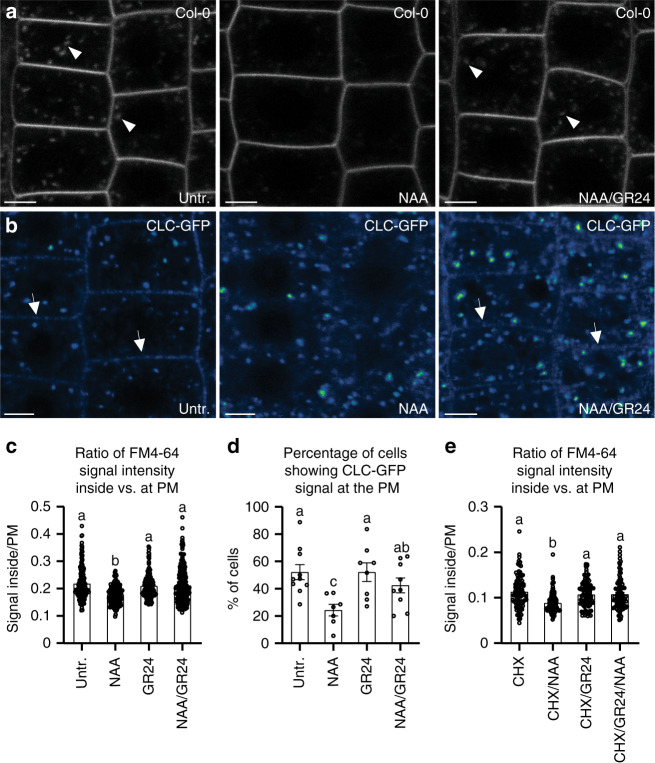
Regulation of auxin-mediated inhibition on endocytosis by SLs in *Arabidopsis*. **a**, **c**, **e** Effect of GR24 on NAA-inhibited FM4-64 uptake. GR24 (1 μM), which alone had no detectable effect on FM4-64 uptake, largely diminished the NAA (10 μM) action of inhibiting FM4-64 uptake (**a**, **c**). Quantitative evaluation of FM4-64 uptake: the quotient between mean fluorescence intensity of the intracellular and PM in the roots was scored (**c** *n* ≥ 150 cells). GR24 treatment also effectively attenuated NAA-mediated inhibition of FM4-64 uptake, when protein synthesis was inhibited by 50 μM cycloheximide (CHX; **e** *n* ≥ 91 cells). Arrowheads indicate endosomal compartments of FM4-64. Scale bars: 5 µm. Data are expressed as mean ± s.e.m. Means with different letters are significantly different at *P* < 0.05 (one-way ANOVA with Fisher LSD test). **b**, **d** Effect of GR24 on NAA-regulated clathrin localization. CLC-GFP distributed at the trans-Golgi network and the PM. NAA (30 μM) treatment induced a transient decrease of the CLC-GFP signal at the PM. GR24 (10 μM), which alone had no detectable effect on CLC-GFP signal, largely prevented NAA action on depletion of CLC-GFP signal from the PM. The percentage of root cells showing CLC-GFP labeling at the PM was scored (**d** *n* ≥ 7 roots). Arrows indicate CLC-GFP distribution at the PM. Scale bars: 5 µm. Data are expressed as mean ± s.e.m. Means with different letters are significantly different at *P* < 0.05 (one-way ANOVA with Fisher LSD test). The above experiments were repeated three times with similar results. Images shown are representative of each treatment. Source data of **c**–**e** are provided in the Source data file.

PIN proteins are internalized by clathrin-mediated endocytosis (CME)^19^ and this endocytic pathway is inhibited by auxin through a TIR1-independent mechanism^16^. Notably, in shoots, SL action has been linked to clathrin-mediated PIN internalization, acting independently of de novo protein synthesis^37^. To gain further insights into the mode of SL action in uncoupling auxin feedback on PIN internalization, we asked whether SLs specifically interfere with the auxin effect on CME in roots. Quantitative evaluation of uptake of the fluorescent endocytic tracer FM4-64^56^ revealed that, as for BFA-induced PIN internalization (Supplementary Fig. 4a, b), FM4-64 uptake itself was not influenced by GR24 treatment (Fig. 4c). However, in contrast, NAA-mediated inhibition of FM4-64 uptake was clearly suppressed in response to GR24 (Fig. 4a, c).

Auxin inhibition of endocytosis coincides with auxin depleting clathrin from the PM. A clathrin light chain fused to GFP reporter protein (CLC-GFP) was found associated with intracellular endomembranes as well as with the PM^57^, and NAA treatment caused a decrease of clathrin-associated fluorescence preferentially at the PM^16^. We then tested the consequence of GR24 treatment, which revealed no visible effect on CLC-GFP localization (Fig. 4d). However, upon co-incubation with NAA, GR24 counteracted NAA-mediated depletion of the PM-localized CLC-GFP, reflected in an efficient recovery of CLC-GFP signal at the PM (Fig. 4b, d). Thus, while SLs alone do not have an obvious effect on PIN internalization, endocytosis or clathrin association with the PM, they specifically interfere with auxin effects on these processes.

Notably, the GR24 effect on auxin-inhibited BFA-induced PIN internalization and FM4-64 uptake could also be observed upon inhibition of de novo protein synthesis (Fig. 4e, Supplementary Fig. 5a–d). We further obtained comparable results that affecting transcription by cordycepin treatments did not influence the ability of GR24, as it was still effective enough to counteract the NAA effect on FM4-64 uptake (Supplementary Fig. 5e) and CLC-GFP depletion from the PM (Supplementary Fig. 5f). Given the involvement of protein ubiquitination in the control of PIN2 endocytic sorting^58^, we determined the 5DS effect on NAA-regulated PIN2 recycling in presence of avadomide and carfilzomib, targeting ubiquitination and proteasome activity, respectively. Both drugs antagonized 5DS effects on NAA-induced inhibition of PIN2 internalization (Supplementary Fig. 5g), highlighting an involvement of protein ubiquitination and proteasome activity in the transmission of the SL effect on the control of auxin feedback on protein endocytosis.

Together, these results are in agreement with, and further extend, previous findings for SL effects on PIN internalization in shoots^37^, as they establish a non-transcriptional SL effect modulating auxin-dependent control of PIN internalization by CME in roots.

### Protein trafficking is linked with auxin canalization

We then asked if the observed SL cellular effects are functionally related with SL regulation of auxin canalization. For this purpose, we tested various mutants defective in CME (*chc2-1* and *chc2-2* alleles) or the auxin effect on PIN trafficking (*doc1* and *big*). Indeed, all these mutants interfered with developmental processes involving canalization, such as leaf venation patterning (Supplementary Fig. 5h), vascular tissue regeneration and formation induced by wounding or external auxin sources^59^. These data strengthen the previously established connection^59^ between subcellular trafficking and their dynamic auxin regulations at the level of individual cells and long-term auxin-induced canalization processes across tissues.

### SL/karrikin signaling interferes with auxin feedback

SLs and karrikins act via MAX2-dependent signaling, and we therefore questioned whether both signals exert long-term effects on vasculature patterning in response to external auxin sources (droplets of auxin in lanolin). IAA application promoted the formation of PIN1-mediated auxin channels and regeneration of vascular strands in *Arabidopsis*, whereas GR24 treatment alone had no influence neither on auxin channel nor on vascular tissue formation (Supplementary Fig. 6a, b). Although SL signaling is essential for lateral bud development, in this experimental set-up, regardless of the presence or absence of lateral buds, GR24 effectively inhibited IAA-induced formation of PIN1-GFP channels and vascular strands from the position where IAA and GR24 were co-applied (Supplementary Fig. 6a, b). Notably, antagonistic effects on auxin channel and vasculature formation were also observed in response to karrikinolide (KAR_1_; Supplementary Fig. 6a, b).

Moreover, we also examined short-term effects of KAR_1_ on auxin feedback at the subcellular level. As in case with SLs, KAR_1_ treatment antagonized NAA-mediated PIN2 lateralization (Supplementary Fig. 6c), and attenuated the NAA effect on BFA-induced PIN2 intracellular accumulation as well (Supplementary Fig. 6d).

Taken together, these data suggest that MAX2-dependent SL and karrikin signaling interfere with auxin feedback control of canalization at the tissue level as well as PIN polarity and trafficking at the cellular level.

## Discussion

Our observations extend the current knowledge about developmental processes regulated by SLs and provide insights into the cellular mechanism of SL action. We show that SLs negatively regulate vascularization of leaves, vasculature regeneration after wounding as well as de novo formation of vasculature from artificial exogenous auxin sources. These processes, together with well-documented effects of SLs on shoot branching, are thought to at least partially depend on the canalization of auxin flow through narrowed auxin-conducting channels that demarcate future vasculature.

Prerequisites for canalization involve the feedback regulation of directional auxin transport, as manifested at the cellular level by the auxin effect on polar, subcellular localization of PIN auxin transporters^4,40^. Our results show that endogenous as well as exogenous SLs interfere with canalization-dependent developmental processes, and specifically interfere with auxin feedback on PIN polarity and clathrin-mediated endocytosis of PIN proteins. This SL action does not require the regulation of transcription and occurs through the known D14- and MAX2-mediated signaling pathways. Thus, SLs may repress a mechanism that enables auxin to inhibit PIN internalization and polarization or SLs may inhibit auxin bioactivity in this cellular context. This is indicated by a proposed role for SLs in the regulation of auxin biosynthesis in context of shoot gravitropism^32^. However, auxin biosynthesis and auxin levels could also be repressed as a consequence of inhibition of auxin transport^60^. Moreover, in our tests, SLs also inhibit the action of exogenously applied auxin, suggesting SLs act downstream of auxin biosynthesis.

It was suggested previously that, in context of shoot branching, SLs destabilize PINs via promoting their internalization from the PM^29,37,61^. However, our observations in roots suggest that SLs do not affect endocytosis or PIN internalization per se, but specifically uncouple the effect of auxin on endocytosis and trafficking processes. Alternatively, SLs could divert endocytic PIN trafficking into an auxin-insensitive pathway, thus making PIN retrieval from membranes more efficient and possibly auxin-insensitive. In any case, given that SLs also interfere with canalization-mediated processes in context of branching and vascular tissue formation and regeneration in shoots, it is likely that the above-mentioned PIN1-GFP-based observations in shoots^29,37,61^ are in fact results of the here-identified SL effects on auxin feedback on PIN internalization.

Our findings identify a cellular mechanism, acting downstream of D14 and MAX2-dependent SL and karrikin signaling, and provide a mechanistic framework for the important part of developmental roles of the pathways, including vascularization and the regulation of root and shoot architecture. Further work should identify the precise molecular links between the SL/karrikin-related pathways and auxin feedback on PIN polarity.

## Methods

### Plant materials

The following transgenic plants and mutants have been described previously: *DR5rev::GFP*^62^; *PIN1::PIN1-GFP*^11^; *CLC::CLC-GFP*^57^; *max1-1*^63^; *DEX*≫*MAX1*, *max1*^29^; *max2-3*^64^; *max4-1*^65^; *d14-1*^66^; *d14-1 htl-3*^42^.

*Arabidopsis* was stably transformed with pTA7002^67^ to only express the empty Dex-inducible GVG cassette. This cassette can occasionally cause unspecific growth and defense defects^44^. However, we observed normal plant growth upon Dex treatment.

### Growth conditions

*Pisum sativum L*. cv. Vladan (Pea) plants were grown in perlite soaked with Richter’s nutrient solution under a 16-h light/8-h dark cycle at 20 °C/18 °C for 7 days. Intact or decapitated (10 mm above the upper bud) plants were used. *Arabidopsis* Columbia ecotype (Col-0) adult plants used for inflorescence stems wounding were individually grown in pots with a soil and vermiculite mixture (1:1, v/v) under a 16 h light/8 h dark cycle at 20 °C for 7–8 weeks. *Arabidopsis* seedlings were grown vertically on half-strength Murashige and Skoog (MS) agar plates under a 16-h light/8-h dark cycle at 21 °C for 4–5 days.

### Drug application and experimental conditions

Exogenous drugs were applied as following: GR24 (*rac*-GR24; 50 mM stock in acetone made freshly; Radboud University Nijmegen or Olchemim) (0.01/0.03/0.09/0.1/1/5/10/20/25/50 µM), (+)-5-deoxystrigol [5DS; 50 mM stock in acetone; Olchemim] (50 μM), karrikinolide (KAR_1_; 10 mM stock in methanol; Toronto Research Chemicals) (10/50 µM), dexamethasone (Dex; 50 mM stock in DMSO; Sigma) (15/50 µM), indole-3-acetic acid (IAA; 10 mM stock in DMSO; Sigma) (0.16/10 µM), 1-naphthaleneacetic acid (NAA; 10 mM stock in DMSO; Sigma) (10/30 µM), BFA (50 mM stock in DMSO; Invitrogen) (25 µM), cycloheximide (CHX; 100 mM stock in DMSO; Sigma) (50 µM), cordycepin (COR; 50 mM stock in DMSO; Sigma) (50 µM), avadomide (Avad; 349.3 mM stock in DMSO; MedChemExpress) (100 µM), or carfilzomib (CFZ; 100 mM stock in DMSO; BioVision) (100 µM). Control treatments contained an equivalent amount of solvent.

For morphological analyses on vein patterning, *Arabidopsis* seedlings were grown on solid MS medium supplemented with GR24. Regarding Dex induction experiments, unless otherwise noted: seedlings were always germinated on medium containing 50 µM Dex. For vasculature regeneration detection, *DEX*≫*MAX1* plants were treated with 15 µM Dex for 5 h by applying Dex directly to the basal parts of inflorescence stems with a brush. For observations on NAA-induced PIN1 relocation, *DEX*≫*MAX1* seedlings were treated with 50 µM Dex on solid medium for 24 h. For testing NAA inhibition on BFA-induced internalization, if not mentioned otherwise: 90 min with 25 µM BFA; or 90 min with 10 µM NAA/BFA co-treatment after 50 min of NAA pretreatment; or 90 min with NAA/5 µM GR24/BFA co-treatment after 50 min of NAA/GR24 pretreatment, in liquid half-strength MS medium. Only for Fig. 3h, i, conditions were slightly different: 60 min with 25 µM BFA; or 30 min pretreatment with 10 µM NAA, followed by 60 min co-treatment of NAA/BFA; or first a 30 min pretreatment with 25/50 µM GR24 or 50 µM 5DS, then another 30 min pretreatment with NAA/GR24 or NAA/5DS, followed by concomitant NAA/GR24/BFA or NAA/5DS/BFA treatment for 60 min. For the other 100% stacked column charts: 60 min with 25 µM BFA; or 30 min pretreatment with 10 µM NAA, followed by 60 min co-treatment of NAA/BFA; or first a 30 min pretreatment with 10 µM NAA, then another 60 min pretreatment with NAA/50 µM GR24, NAA/50 µM 5DS or NAA/50 µM KAR_1_, followed by concomitant NAA/GR24/BFA, NAA/5DS/BFA or NAA/KAR_1_/BFA treatment for 60 min. For other BFA related visualization, seedlings were treated by 60 min with 25 µM BFA; or 60 min with 5 µM GR24/BFA co-treatment after 30 min of GR24 pretreatment. For inhibition of de novo protein synthesis in BFA related visualization, pretreatments of 30 min with 50 µM CHX were always applied beforehand. For inhibition of ubiquitination and proteasomal degradation in BFA related visualization, pretreatments of 80 min with 100 µM Avad or 100 µM CFZ were always applied beforehand. For evaluating NAA-induced PIN relocation, seedlings were treated by 10 μM NAA for 4 h; 50 µM GR24, 50 µM 5DS, or 50 µM KAR_1_ for 4 h; or NAA/GR24, NAA/5DS, or NAA/KAR_1_ for 4 h following 1 h of GR24, 5DS, or KAR_1_ pretreatment. For observation on FM4-64 uptake, seedlings were treated by 10 µM NAA, 1 µM GR24, or NAA/GR24 for 80 min, respectively. For observation on CLC-GFP abundance at the PM, seedlings were treated by 30 µM NAA, 10 µM GR24, or NAA/GR24 for 80 min, respectively. For inhibition of de novo protein synthesis or transcription in both experiments, 50 µM CHX or 50 µM COR was always applied together with NAA, GR24, or NAA/GR24.

### Auxin transport assays in pea

For auxin transport assay on the axillary buds, the upper axillary buds were treated with water lanolin pastes or pastes with GR24 (0.03 μM). After 4 h, the treated and untreated plants were decapitated 10 mm above the upper bud. 0.5 μl of [5-^3^H]-IAA (American Radiolabeled Chemicals, 925 Gbq mmol^−1^, 6666 Bq μl^−1^) diluted in 50% ethanol was then applied to the tip of the axillary buds after decapitation in 6 h. Following 1.5 h treatment, the stems at a distance of 0–4 and 4–8 mm below the upper axillary buds were cut into 4 mm segments, respectively. All samples were incubated in a dioxane-based liquid scintillator cocktail overnight. The [^3^H] activity was then measured with a scintillation spectrophotometer Packard TRI/Carb 2000 (Packard).

### Gene expression analyses

For gene expression of *PsDRM1*, GR24 (0.03 μM) in water lanolin paste was applied on the upper axillary bud of decapitated plants as a ring. *PsDRM1* expression was then followed in the untreated lower and treated upper axillary buds.

Total RNA was extracted from buds of pea plants using the RNeasy Plant Mini Kit (Qiagen). RNase-free DNase I (Qiagen) was used to remove genomic DNA. RNA was then reverse transcribed using the Superscript III cDNA kit (Invitrogen). Resulting cDNAs were used to detect *PsDRM1* gene expression by quantitative Real-Time PCR (qRT-PCR) using LC 480 SYBR Green I Master Mix (Roche Diagnostics) with the specific primers^40^ (Supplementary Table 1). The gene expression normalization was performed by using the combination of three reference genes (*Psβ-tubulin*, *PsActin*, and *PsEF1-α*).

### Vascular tissue formation analyses

Young *Arabidopsis* plants with inflorescence stems having primary tissue architecture (vascular bundles separated by interfascicular parenchyma sectors) were used for analyzing vasculature regeneration and formation after wounding or from local application of compounds according to method described in previous study^41,68^. Briefly, first step was aimed to obtain a closed ring of active vascular cambium and secondary tissue architecture in immature inflorescence stems; second step was to analyze regeneration of incised vascular cambium and formation of new vessels in wounded stems.

### In situ expression and localization analyses

In *Arabidopsis*, whole-mount immunolocalization was performed following the published protocol^69^. Antibodies were diluted as follows: 1:1000 for rabbit anti-PIN1^15^ (produced and processed in lab); 1:1000 for rabbit anti-PIN2^70^ (produced and processed in lab); and 1:600 for CY3-conjugated anti-rabbit secondary antibody (Sigma, C2306). In pea, water lanolin pastes containing IAA (0.16 μM), or IAA/GR24 (0.16 µM/0.09 µM) were applied on the stem stump or on the stem 2 mm below lateral incision. Immunolocalization was performed on longitudinal pea stem segments as described for *Arabidopsis* stem^69^. The *Arabidopsis* anti-PIN1 antibody can also recognize the homologous PIN protein in pea^4^. Antibodies were diluted as follows: 1:1000 for rabbit anti-PIN1^15^ (produced and processed in lab); and 1:500 for CY3-conjugated anti-rabbit secondary antibody (Sigma, C2306). All the fluorescence signals were evaluated on Zeiss LSM 700, Zeiss LSM 710, Zeiss Observer. Z1, Leica TCS SP2, Olympus Fluoview FV1000, or Olympus Fluoview 200 confocal scanning microscopes. Unless otherwise noted, the same microscope settings were usually used for each independent experiment and pixel intensities were taken into account when comparing the images between different samples. Images were finally assembled in Adobe Photoshop CC 2015 and Adobe Illustrator CS6.

## Supplementary information

Supplementary Information

Reporting Summary

## Data Availability

The data supporting the findings of this study are available within the paper and its supplementary information files, or from the corresponding authors upon reasonable request. The source data underlying Figs. 2c–e, 3b–d, f–h, 4c–e, and Supplementary Figs. 1b–d, 2b, 3b, d, 4a, b, e, f, i–k, 5c–h, 6b–d are provided as a Source data file.

## Source data

Source Data
