# Supplementary material for: Strigolactones inhibit auxin feedback on PIN-dependent auxin transport canalization
Source: Nat Commun. 2020 Jul 14;11:3508. doi: 10.1038/s41467-020-17252-y (PMC7360611; doi:10.1038/s41467-020-17252-y)
Supplement: Supplementary file 1 — Supplementary Information [file 41467_2020_17252_MOESM1_ESM.docx]

**Supplementary Information**

**Strigolactones inhibit auxin feedback on PIN-dependent auxin transport canalization**

**Zhang *et al.***

**
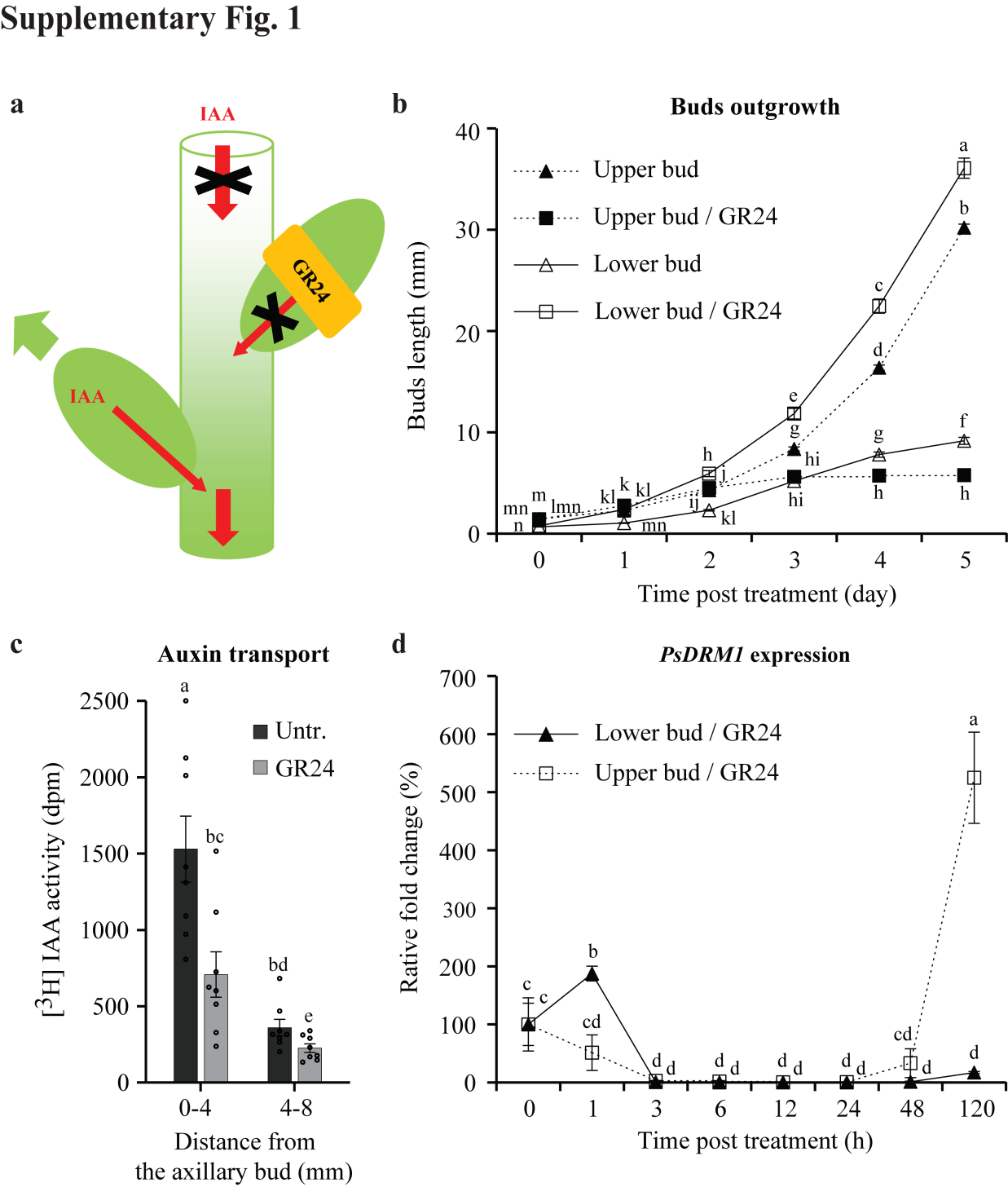
**

**Supplementary Fig. 1** SL effects on dormancy and auxin transport in pea. **a** Scheme representing decapitated plants treated with the synthetic SL analog GR24 in a ring on the upper bud. Red arrows represent auxin (IAA) flow. Red arrows crossed with black X represent inhibited auxin flow. Green arrow represents bud outgrowth. Outgrowth of the upper bud, which is normally released from dormancy after decapitation, is inhibited by GR24 treatment, leading to the development of the lower bud instead. **b** Outgrowth of the axillary buds on the decapitated plant: untreated or upper buds treated with GR24 (0.03 µM). Untreated upper buds showed continuous outgrowth, whereas GR24 application allowed a short period of outgrowth for the treated buds but inhibits further outgrowth. The lower buds behaved oppositely to the upper buds, such that when the upper buds were growing, outgrowth of the lower buds were inhibited, and only after inhibition of outgrowth of the upper buds by GR24 application do the lower buds started to grow. Data are expressed as mean ± SE (*n* ≥ 26 buds). Means with different letters are significantly different at *P* < 0.05 (One-way ANOVA with Fisher LSD test). **c** [^3^H]-IAA transport applied on the upper axillary bud. Transport was measured in two segments of stem at a distance of 0-4 mm and 4-8 mm below the upper axillary bud. Application of GR24 (0.03 µM) decreased auxin transport from the bud. This contrasted with other results showing no effect of GR24 on [^3^H]-IAA transport^1^, but in that case outgrowing buds were examined. Data are expressed as mean ± s.e.m. (*n* = 8 plants). Means with different letters are significantly different at *P* < 0.05 (One-way ANOVA with Fisher LSD test). **d** Relative expression of *PsDRM1* in the pea axillary buds. The bud growth and transport observations were matched by bud activity status, as confirmed by the expression of the dormancy marker gene *DORMANCY-ASSOCIATED PROTEIN1* (*PsDRM1*). Gene expression was detected in the lower and upper axillary buds of decapitated plants treated with GR24-ring (0.03 µM) on the upper buds. Data are expressed as mean ± SE of two biological replicates. Means with different letters are significantly different at *P* < 0.05 (One-way ANOVA with Fisher LSD test). Total RNA for each sample was isolated from 30 buds. Four technical replicates were analyzed in each independent experiment. The above experiments were repeated at least twice with similar results. Source data of b-d are provided in the Source Data file


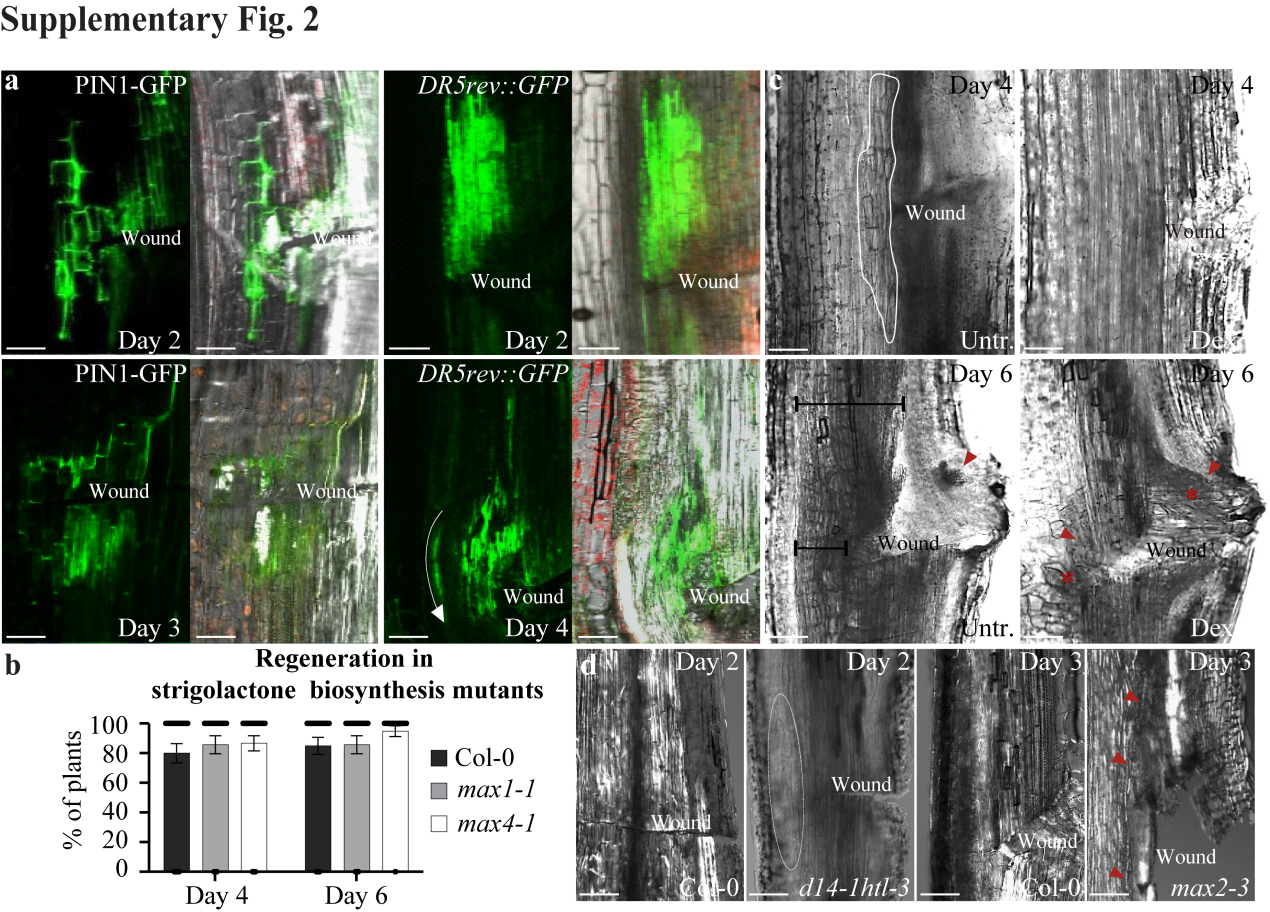


**Supplementary Fig. 2** SL effect on vasculature regeneration after wounding in *Arabidopsis.* **a** Cellular events related to auxin canalization and vascular tissue development after wounding: initial broad and apolar PIN1 expression around the wound and auxin response accumulation (*DR5rev::GFP*) above the wound at 2 days after wounding; establishment of narrow and polarized PIN expressing at the day 3 and auxin-conducting channels circumventing the wound beginning from the day 4. Arrows indicate auxin channels. Right panels are merged images of fluorescence and light transmitted signals. Scale bars: 100 µm. **b** Vasculature regeneration in SL biosynthesis-defective mutants *max1-1* and *max4-1*. Temporal changes during vascular tissue reconstruction in *max1-1* and *max4-1* were comparable to that of the wild type. First vessels around a wound appeared at the day 4 and completely continued vessel strands developed at the day 6. However, the regenerated vasculature of *max1-1* and *max4-1* was more abundant as compared to wild-type control (see main Fig. 2b). Data are expressed as mean ± s.e.m. (*n* ≥ 35 inflorescence stems). **c** Vasculature regeneration in *DEX>>MAX1* plants. In the untreated *DEX>>MAX1*, vascular tissue regenerated around a wound at the day 4 and the layer of regenerated vessels enlarged at the day 6. In contrast, there was no regenerated vasculature around the wound formed in Dex-induced *DEX>>MAX1* stems. Outline indicates developed vasculature. The line segments indicate thickness of regenerated vasculature above and around a wound. Arrowheads indicate the vessel-like cells differentiated from callus. Asterisks indicate extended callus. Scale bars: 100 µm. **d** Vascular tissue regeneration in SL/karrikin signaling-defective mutants *d14-1htl-3* and *max2-3*. Vasculature regeneration in wounded stems of *d14-1htl-3* and *max2-3* occurred faster than that in the wild type. The differentiated vessels initiated in *d14-1htl-3* and *max2-3* at the day 2 and the day 3 after wounding, respectively, whereas there was no regeneration in wild-type stems at these time points. Circle indicates a group of vessel-like cells differentiated around a wound. Arrowheads indicate differentiated vasculatures. Scale bars: 100 µm. The above experiments were repeated twice with similar results. Images shown are representative of each treatment. Source data of b are provided in the Source Data file


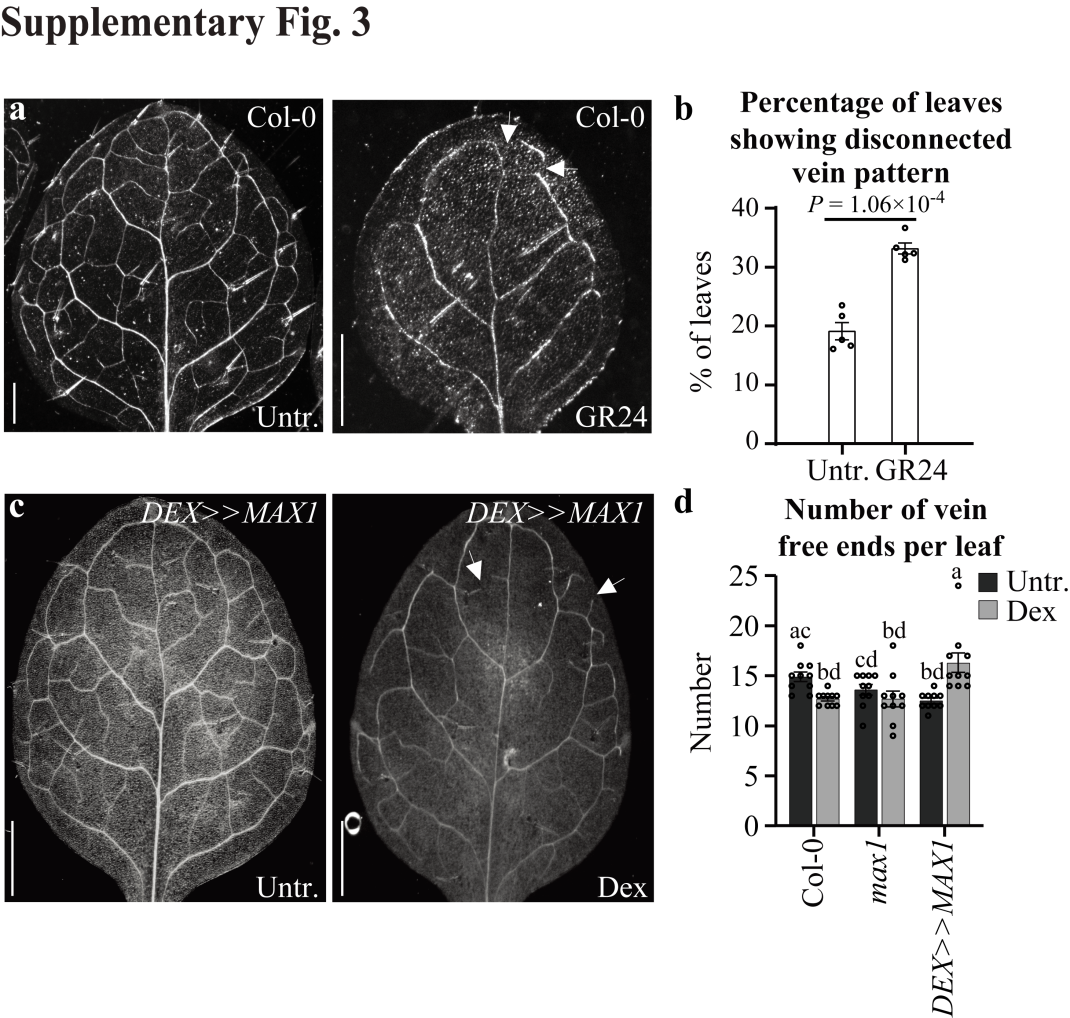


**Supplementary Fig. 3** SL effect on vein patterning in *Arabidopsis*. **a**, **b** Effect of exogenous SLs on vein patterning in primary leaves. Wild-type leaves germinated on media supplemented with GR24 (20 µM) showed suppressed vascular network and disconnected vein pattern (**a**). Quantification of irregular vein pattern: GR24 treatment inhibited the formation of continuous veins (**b**; *n* ≥ 192 leaves). Scale bars: 0.5 mm. Data are expressed as mean ± s.e.m. of five biological replicates. *P* value was calculated using Welch’s two-tailed *t*-test. **c**, **d** Effect of endogenous SLs on vein patterning in primary leaves. *DEX>>MAX1* plants germinated on 50 µM Dex, inducing SL biosynthesis, developed more simplified vasculature with more free ends. Data are expressed as mean ± s.e.m. (*n* = 10 leaves). Means with different letters are significantly different at *P* < 0.05 (One-way ANOVA with Fisher LSD test). Scale bars: 0.5 mm. The above experiments were repeated at least three times with similar results. Images shown are representative of each treatment. Source data of b and d are provided in the Source Data file


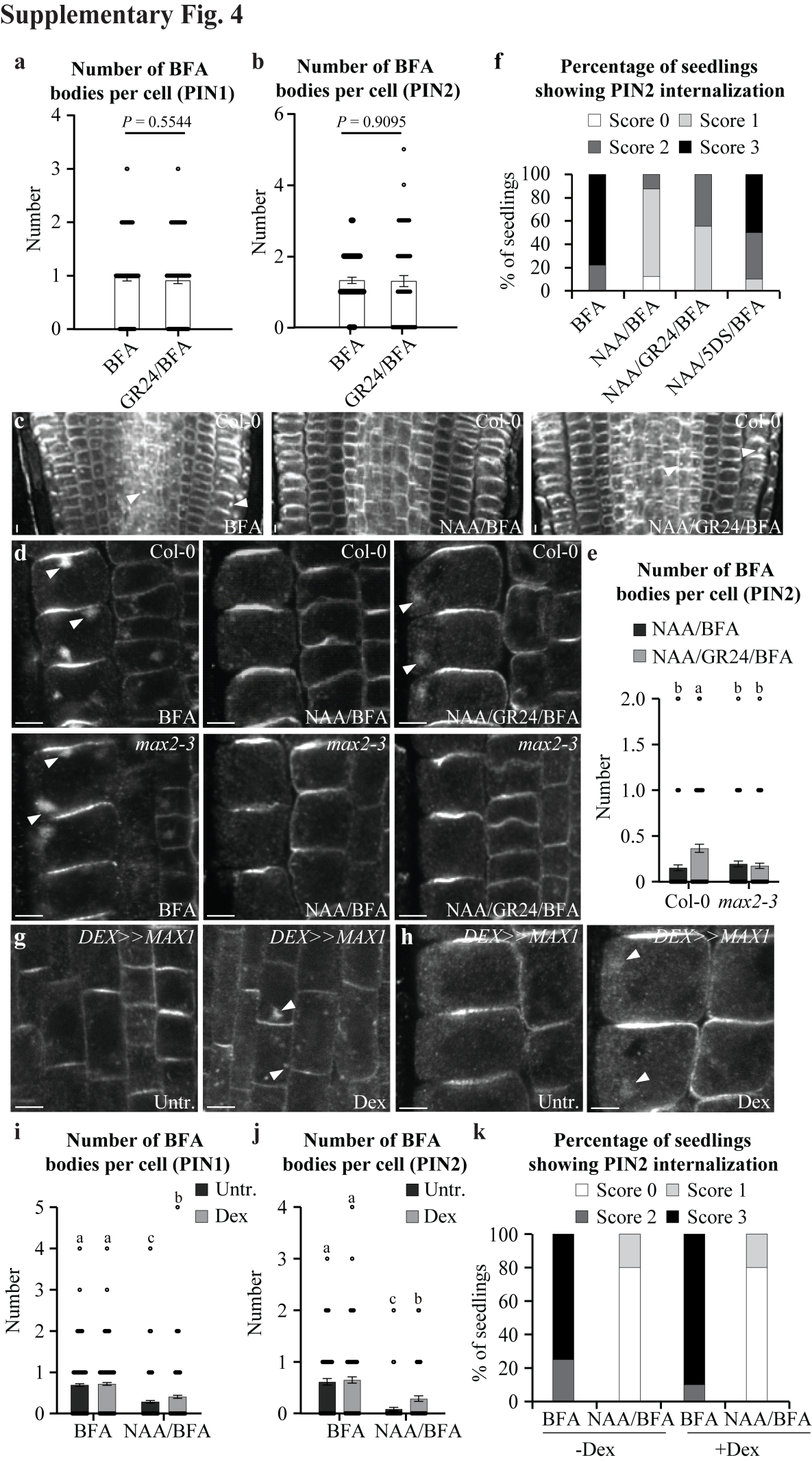


**Supplementary Fig. 4** SL effect on PIN subcellular dynamics in *Arabidopsis.* **a**, **b** No effect of GR24 alone on BFA-visualized PIN internalization. BFA (25 µM)-induced PIN1 and PIN2 internalization was not influenced by 5 µM GR24, which was sufficient to attenuate the NAA effect in wild type. The number of BFA bodies in PIN1 (**a**) and PIN2 (**b**) per root cell was scored (*n* ≥ 67 cells). Data are expressed as mean ± s.e.m. *P* values were calculated using Welch’s two-tailed *t*-tests. **c**-**e** Effect of GR24 on NAA-mediated inhibition of BFA-visualized PIN internalization. Immunolocalization of PIN1 (**c**) and PIN2 (**c**, **d**) in root cells: BFA-induced internalization of PIN1 and PIN2 was inhibited by NAA (10 µM) and this inhibitory effect of NAA was partially counteracted by GR24 at 50 µM (**c**) in wild-type roots. Lower dose of GR24 at 5 µM had the same effect (**d**) as in (**c**). Overview pictures of PIN1 and PIN2 internalization are in **c** and close up pictures of PIN2 are in **d**. This competitive effect of GR24 (5 µM) on NAA action was reduced in the *max2-3* mutant (**d**). Arrowheads indicate PIN proteins internalized into BFA compartments. Scale bars: 5 µm. The number of PIN2-containing BFA bodies per root cell in NAA/BFA- or NAA/GR24/BFA-treated wild-type and *max2-3* mutant seedlings was scored (**e**; *n* ≥ 161 cells). Data are expressed as mean ± s.e.m. Means with different letters are significantly different at *P* < 0.05 (One-way ANOVA with Fisher LSD test). **f** Interference with NAA effect on PIN2 internalization mediated by synthetic SL GR24 and natural SL 5DS. The roots (*n* ≥ 8) were scored blind and the percentage of roots displaying almost undetectable (Score 0), weak (Score 1), stronger (Score 2), or very severe (Score 3) PIN2 internalization was determined. **g**-**k** Endogenous SLs-mediated interference with NAA-inhibited BFA-visualized PIN internalization. Immunolocalization of PIN1 and PIN2 in root cells: BFA-induced internalization of PIN1 (**g**) and PIN2 (**h**) was inhibited by NAA in root cells of non-induced control, but this effect was attenuated in 50 µM Dex induced *DEX>>MAX1* plants. Arrowheads indicate PIN proteins internalized into BFA compartments. Scale bars: 5 µm. The number of PIN1- and PIN2-containing BFA bodies per root cell was scored, respectively (**i, j**; *n* ≥ 111 cells). Data are expressed as mean ± s.e.m. Means with different letters are significantly different at *P* < 0.05 (One-way ANOVA with Fisher LSD test). Dex-treated transgenic plants expressing the GVG system alone did not give significant phenotype in terms of auxin-mediated PIN2 trafficking, compared to the non-induced controls (**k**). The roots (*n* ≥ 8) were scored blind and the percentage of roots displaying almost undetectable (Score 0), weak (Score 1), stronger (Score 2), or very severe (Score 3) PIN2 internalization was determined. The above experiments were repeated three times with similar results. Images shown are representative of each treatment. Source data of a, b, e, f, and i-k are provided in the Source Data file

**
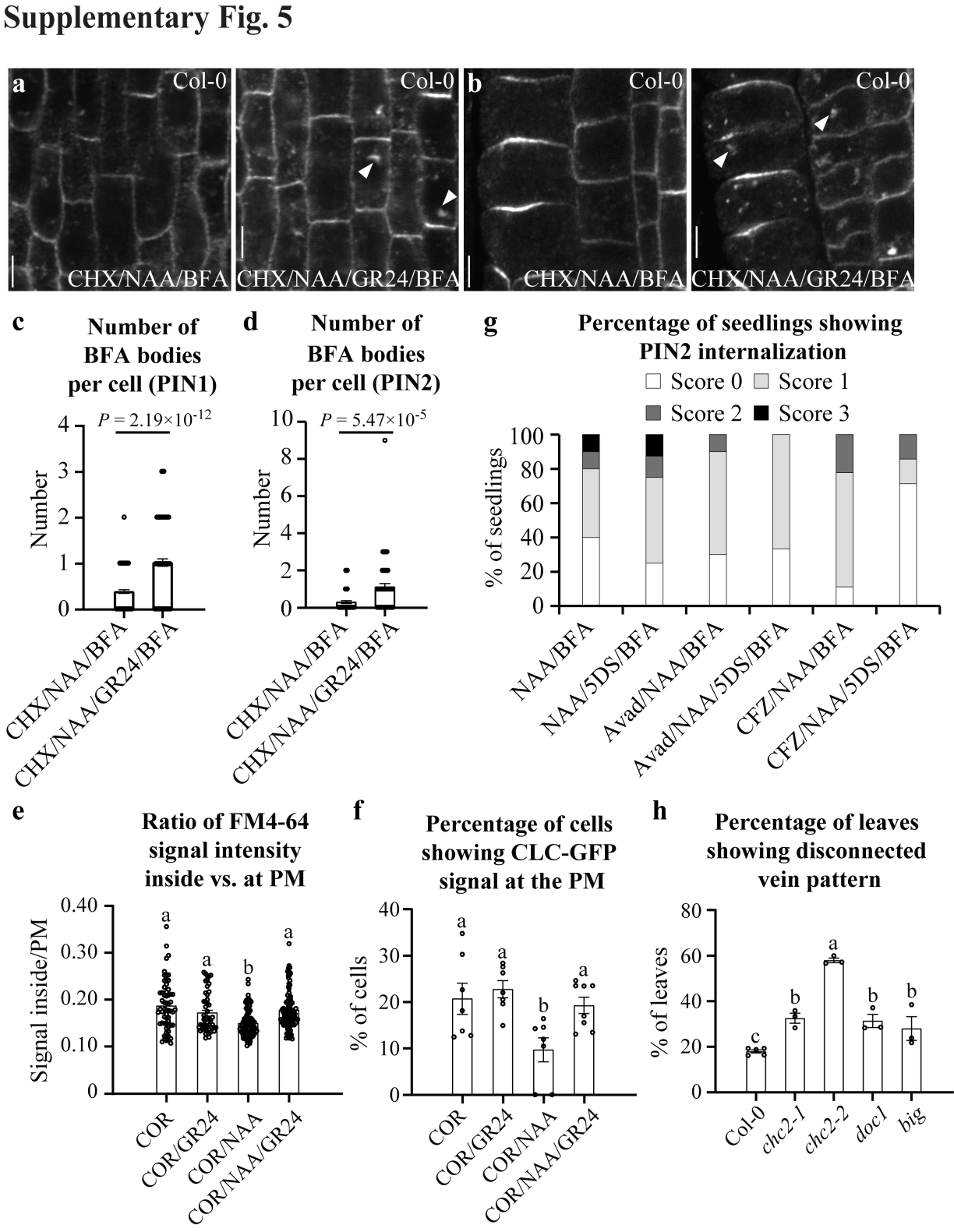
Supplementary Fig. 5** Non-transcriptional SL effect on PIN subcellular dynamics in *Arabidopsis.* **a**-**d** Non-transcriptional effect of GR24 on NAA inhibition of BFA-induced PIN internalization. GR24 treatment effectively attenuated NAA-mediated inhibition of PIN1 (**a**) and PIN2 (**b**) internalization when protein synthesis was inhibited by CHX (50 µM). Scale bars: 5 µm. The number of BFA bodies in PIN1 (**c**) and PIN2 (**d**) per root cell was scored (*n* ≥ 56 cells). Data are expressed as mean ± s.e.m. *P* values were calculated using Welch’s two-tailed *t*-tests. **e**, **f** Non-transcriptional effect of GR24 on NAA-mediated endocytosis and CLC abundance at the PM. GR24 treatment effectively attenuated NAA-mediated inhibition of FM4-64 uptake when transcription was inhibited by cordycepin (COR; 50 µM; **e**). The quotients between FM4-64 mean fluorescence intensity of the intracellular and PM in the roots were scored (**e**; *n* ≥ 50 cells). GR24 treatment antagonized NAA-mediated depletion of CLC-GFP from the PM in presence of COR (**f**). The percentage of root cells showing CLC-GFP labelling at the PM was scored (**f**; *n* ≥ 7 roots). Data are expressed as mean ± s.e.m. Means with different letters are significantly different at *P* < 0.05 (One-way ANOVA with Fisher LSD test). **g** Quantification of PIN2 internalization in roots. The inhibitory effect of NAA on BFA-induced PIN2 internalization was attenuated by 5DS (50 μM) in wild-type roots. Application of avadomide (Avad; 100 µM) or carfilzomib (CFZ; 100 µM) could prevent the 5DS effect. The roots (*n* ≥ 7) were scored blind and the percentage of roots displaying almost undetectable (Score 0), weak (Score 1), stronger (Score 2), or very severe (Score 3) PIN2 internalization was determined. **h** Vasculature patterning in mutants interfering with CME (*chc2-1* and *chc2-2* alleles) and auxin effect on PIN trafficking (*doc1* and *big*). The overall frequency of irregular vein formation was markedly increased in these mutants as compared to that of wild type (*n* ≥ 57 leaves). Data are expressed as mean ± s.e.m. Means with different letters are significantly different at *P* < 0.05 (One-way ANOVA with Fisher LSD test). The above experiments were repeated at least twice with similar results. Images shown are representative of each treatment. Source data of c-h are provided in the Source Data file


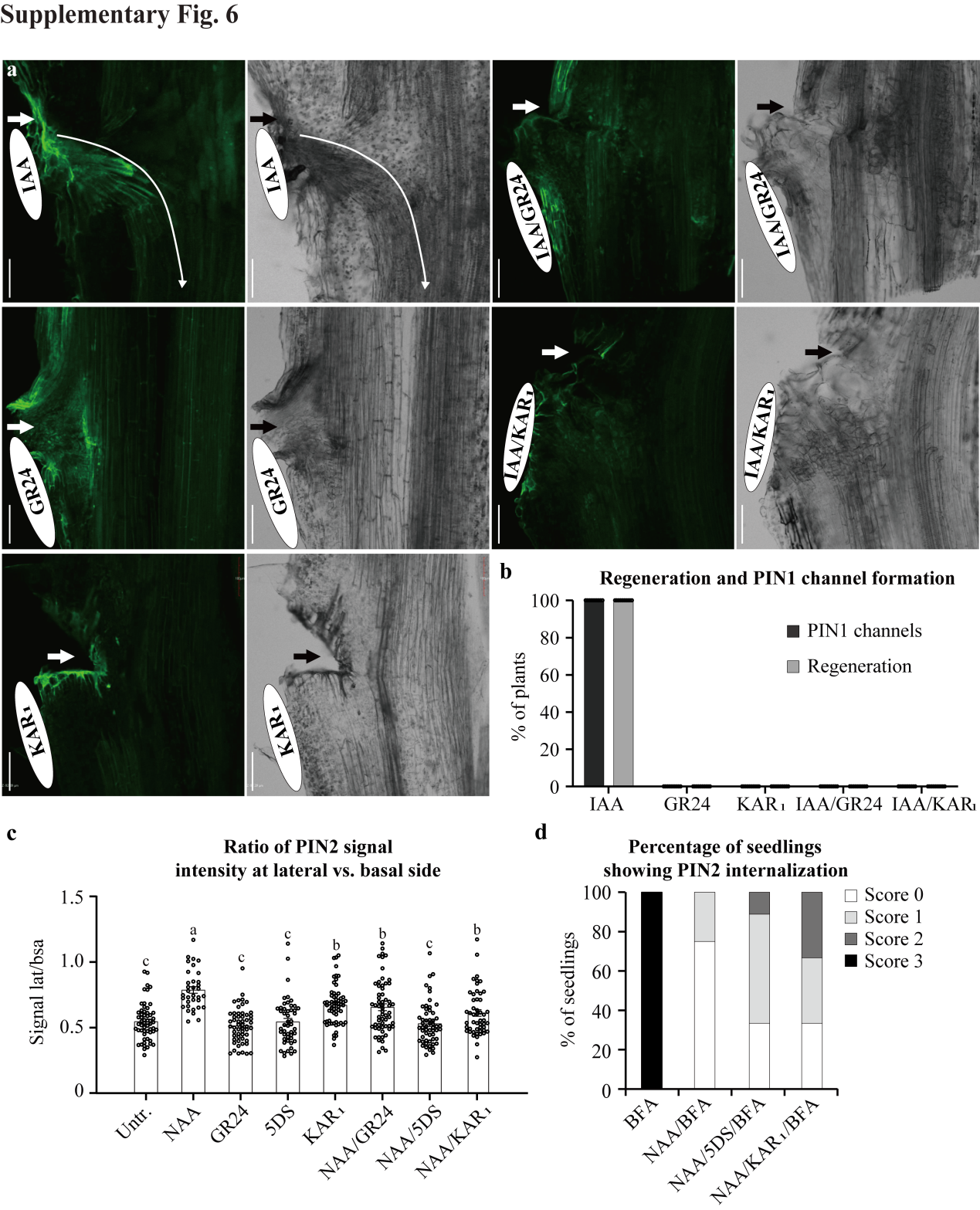


**Supplementary Fig. 6** SL/karrikin signaling-mediated interference of auxin feedback at both tissue and cellular levels. **a**, **b** Auxin canalization and vascular strand formation in *Arabidopsis* stems. Chemicals were locally applied in droplets of lanolin pastes (indicated by white ovals) below the wounds (indicated by arrows). Both GR24 (10 μM) and KAR_1_ (10 μM) treatments inhibited IAA (10 μM)-mediated vasculature regeneration and auxin channels formation (**a**). Both the fluorescence and light transmitted images are shown. Curved lines indicate formed PIN1 channels and regenerated vasculature. Scale bars: 100 µm. Vasculature regeneration and PIN1 channels formation were scored (*n* = 10 inflorescence stems) (**b**). **c** Quantification of auxin-mediated PIN2 lateralization in root cortex cells. Exogenously applied GR24 (50 μM), 5DS (50 μM), or KAR_1_ (50 µM) attenuated NAA effect on PIN2 lateralization in wild-type roots. Ratio between mean fluorescence intensity of the lateral and basal membrane in young cortex cells were scored (*n* ≥ 34 cells). Data are expressed as mean ± s.e.m. Means with different letters are significantly different at *P* < 0.05 (One-way ANOVA with Fisher LSD test). **d** Quantification of PIN2 internalization in roots. BFA-induced PIN2 internalization was inhibited by NAA. This inhibitory effect of NAA was partially counteracted by 5DS (50 μM) and KAR_1_ (50 μM) in wild-type roots. The roots (*n* ≥ 8) were scored blind and the percentage of roots displaying almost undetectable (Score 0), weak (Score 1), stronger (Score 2), or very severe (Score 3) PIN2 internalization was determined. The above experiments were repeated at least twice with similar results. Images shown are representative of each treatment. Source data of b-d are provided in the Source Data file

**Supplementary Table 1.** Primers used for qRT-PCR.

| **Primer name** | **Primer sequence (5'-3')** |
| --- | --- |
| *PsDRM1*-F | AACTCACCACCACCCTCAAAGATG |
| *PsDRM1*-R | GATGTAGACACGTGGCAGAAGATG |
| *Psβ-tubulin*-F | GCTCCCAGCAGTACAGGACTCT |
| *Psβ-tubulin*-R | TGGCATCCCACATTTGTTGA |
| *PsActin*-F | TGTCAGCCACACTGTCCCTATC |
| *PsActin*-R | CAAGACGAAGGATGGCATGT |
| *PsEF1-α*-F | CTGTCATGGATGCACCT |
| *PsEF1-α*-R | TCCTTAGAGATACCGGCTT |

**Supplementary Reference**

1. Brewer, P. B., Dun, E. A., Ferguson, B. J., Rameau, C. & Beveridge, C. A. Strigolactone acts downstream of auxin to regulate bud outgrowth in pea and *Arabidopsis*. *Plant Physiol.* **150**, 482–493 (2009).
